# Narrow Band Region-Based Active Contours Model for Noisy Color Image Segmentation

**DOI:** 10.1155/2014/307416

**Published:** 2014-07-10

**Authors:** Xiaomin Xie, Aijun Zhang, Changming Wang, Xiangfei Meng

**Affiliations:** School of Mechanical Engineering, Nanjing University of Science and Technology, Nanjing, Jiangsu 210094, China

## Abstract

A narrow band active contour model for color image segmentation is proposed, which applies local statistics to improve the robustness against noise. The crux of our approach is to use intensity mean of local region to define the force function within a level set framework, within which a narrow band is implemented to further improve the computational efficiency. In addition, the image is segmented from channel-to-channel, which shows superior performance when the intensities of the object and background are similar. Furthermore, a multichannel segmentation combination method is used to integrate the information of multiple level sets. The proposed model has been applied to both synthetic and real images with expected results, and the comparison with the state-of-the-art approaches demonstrates the accuracy and superiority of our approach.

## 1. Introduction

Image segmentation is a process that divides the image into meaningful parts. It plays a vital role in the field of image analysis and pattern recognition. Up to now, there have been kinds of algorithms for image segmentation, such as thresholding [1], clustering [2], and active contour models [3–9].

Active contour models (ACMs) have been used widely and successfully in image segmentation [3–16]. The basic idea is to make a contour deform so as to minimize a given energy function and generate desired segmentation [10]. ACMs can be categorized into two classes: edge-based [3–5] and region-based ACMs [6–16].

The region-based models [6–16] utilize some region descriptors such as color and intensity, showing better performance over the edge-based models for images with noise and weak boundaries in most cases. One of the most famous region-based models is CV model [6], which assumes that each region of the image is statistically homogeneous. Then, it is extendable to a two-phase model [7] for vectorial images and the multiphase model [8, 9] which partitions image into arbitrary parts. The energy function of CV model is measured by the difference between each pixel and the region intensity means. However, the CV model, as well as its extension, fails to detect the object boundaries accurately in the presence of intensity nonuniformity and noise. Aimed at the problems caused by intensity inhomogeneities, some local region-based models are proposed [10–12] in the literature, which draw upon intensity information in spatially varying local regions determined by a scale parameter [11, 12]. By sliding the Gaussian kernel to each region found by the contour, the local region model approximates intensity averages at a certain scale. According to the level set function, the local region-based models are classified into signed distance function (SDF) ACMs [10] and local approximation SDF ACMs [12, 13]. For SDF ACMs, the level set function is defined as a SDF. And thus the reinitialization is required in this evolution. In order to improve the efficiency, only values of the level set function on a narrow band around the zero level set are calculated [10].

Usually, color images are processed by converting them into scalar ones. The models in [7, 17, 18] are similar to the approaches which transform color images into scalar ones. When the objects have similar intensities, they are invalid after the application of averaging all the channels [14]. In [14], the classical CV model is extended for color images, which is based on the idea of segmenting an image from channel-to-channel and then combining the segmentation results. The extended scheme provides better capacity of color discrimination and segmentation accuracy. However, when the image is corrupted by noise, the extended scheme starts to suffer from difficulties.

In this paper, a region-based ACM to segment noisy color images in a much more efficient manner is proposed. The idea of incorporating localized statistics into a variational framework, proposed firstly by Lankton and Tannenbaum [10], makes contribution to the robustness to noise [15]. The proposed model measures both the global and local statistical properties of the object to adjust the level set function and fit the object with the zero level set, which is found to improve the robustness to strong noise and hold global dependence greatly. Meanwhile, drawing inspiration from the works in [14], we segment color images from channel-to-channel. Furthermore, a combination method is used to integrate the segmentation results in each channel. Thus, the proposed approach possesses the ability of handling with close intensity images. We define the energies in terms of a signed distance function. In order to improve efficiency, a narrow band [10, 16] is used around the contour, and the deformations of local intensity means in the local window are confined within this band.

The rest of the paper is organized as follows. Some related models for both gray and color images are briefly reviewed in Section 2. Then, Section 3 describes how the proposed model is established. Afterwards, the implementation and experiment results are presented and analyzed in Section 4. And then the conclusion is conducted in the last section.

## 2. Related Works

Let *I* : Ω → *R* ^*d*^ be a given vector valued image, and let Ω ⊂ *R* ^*n*^ be the image domain. *d* ≥ 1 is a vector dimension of the vectorial image *I*(*x*). For gray images, *d* = 1, and for color images, *d* = 3. *x* of *I*(*x*) is a pixel in Ω. The goal of image segmentation is to divide the image into disjoint subregions Ω_1_,…, Ω_*N*_.

### 2.1. Local Region-Based Framework [10]

Lankton and Tannenbaum propose a natural framework that allows any region-based energy to be localized in a fully variational way [10]. Let *C* denote a closed contour, which is represented as the zero level set of the level set function *ϕ*; that is, *C* = {*x*∣*ϕ*(*x*) = 0, *x* ∈ Ω}. The level set function (LSF) is defined as a signed distance function. We employ the following approximation of the smoothed Heaviside function to specify the interior of *C*:

(1) $$\begin{array}{c} H\left(\phi \left(x\right)\right)=\{\begin{array}{cc} 1 & \phi <-\varepsilon \\ 0 & \phi >\varepsilon \\ \frac{1}{2}\left\{1+\frac{\phi }{\varepsilon }+\frac{1}{\pi }\sin\left(\frac{\pi \phi \left(x\right)}{\varepsilon }\right)\right\} & \text{otherwise}. \end{array} \end{array}$$

Similarly, the exterior of *C* is defined as (1 − *H*(*ϕ*(*x*))). To specify the area just around the curve, a smoothed version of the Dirac function is defined as

(2) $$\begin{array}{c} \delta \left(\phi \left(x\right)\right)=\{\begin{array}{cc} 1 & \phi =0\\ 0 & \left|\phi \right|<\varepsilon \\ \frac{1}{2\varepsilon }\left\{1+\cos\left(\frac{\pi \phi \left(x\right)}{\varepsilon }\right)\right\} & \text{otherwise}. \end{array} \end{array}$$

The parameter *ε* is usually set to 1.5 as in [4, 5]. Then *B*(*x*, *y*) is used to mask local regions:

(3) $$\begin{array}{c} B\left(x,y\right)=\{\begin{array}{cc} 1, & ||x-y||<r\\ 0, & \text{otherwise}. \end{array} \end{array}$$

This function will be 1 when the point *y* is within a ball of radius *r* centered on *x*, and 0 otherwise. In order to keep the curve smooth, we penalize the length of the curve weighted by a parameter *ν*. Using *B*(*x*, *y*), the energy functional based on a generic force function *F* is written as

(4) F(ϕ)=∫Ωxδ(ϕ(x))∫ΩyB(x,y)·F(I(y),ϕ(y))dy dx +ν∫Ωxδ(ϕ(x))|∇ϕ(x)|,

where *F* refers to a generic internal energy measure, which can be chosen mainly according to the images to be segmented, such as uniform modeling (UM) energy, mean separation (MS) energy, or histogram separation (HS) energy [10, 16].

Taking the first variation of the energy function with respect to *ϕ*, we obtain the following gradient flow:

(5) ∂ϕ∂t(x)=δ(ϕ(x))∫ΩyB(x,y)·∇ϕ(y)F(I(y),ϕ(y))dy +νδ(ϕ(x))div⁡(∇ϕ(x)|∇ϕ(x)|).

### 2.2. Vectorial Multiphase Chan-Vese Model [7]

Reference [7] provides an extended multiple model of the Chan-Vese model [6] for gray images to the vector valued case. In level set form, for *m*-level sets, there are *M* = 2^*m*^ phases that partition the image into *M* regions. *I* ^*i*^(*x*)  (*i* = 1,2, 3) is the *i*th channel of the color image *I*(*x*). Each channel contains the same image with different colors. The model is defined as

(6) E(ϕ)=∬Ω13·∑i=13(Ii(x,y)−c11i)2H(ϕ1)H(ϕ2)dx dy +∬Ω13·∑i=13(Ii(x,y)−c12i)2H(ϕ1)(1−H(ϕ2))dx dy +∬Ω13·∑i=13(Ii(x,y)−c21i)2(1−H(ϕ1))H(ϕ2)dx dy +∬Ω13·∑i=13(Ii(x,y)−c22i)2(1−H(ϕ1))       ×(1−H(ϕ2))dx dy +∬Ω(|∇H(ϕ1)|+|∇H(ϕ2)|)dx dy,

where *c*_11_^*i*^, *c*_12_^*i*^, *c*_21_^*i*^, and *c*_22_^*i*^ are the average intensities of the regions in the *i*th channel.

The CV-like models [6–9, 14] are based on the differences between the intensity of each pixel and the global intensity means. Benefited from the global dependence, the CV-like models are adequate in the presence of noise to some extent [6, 7]. However, when the noise level is high, they begin to suffer from difficulties. In addition, since the vectorial CV model uses the weighted average method to average all the channels, it is difficult to distinguish the objects.

### 2.3. Extended Scheme of Chan-Vese Model for Color Image Segmentation [14]

In [14], the classical CV model [6] is generalized for color images by segmenting an image from channel-to-channel (STP-CV model for short). The energy function for every channel is defined as

(7) Ei(c1i,c2i,Ci)=μi length(Ci)+νiArea(Ω1i) +λ1i∬Ω1i|Ii(x,y)−c1i|2dx dy +λ2i∬Ω2i|Ii(x,y)−c2i|2dx dy,

where *C* ^*i*^ is the curve in the *i*th channel. *c*_1_^*i*^ and *c*_2_^*i*^ are the means inside and outside of *C* ^*i*^, respectively. *μ* ^*i*^ and *ν* ^*i*^ are constants. *λ*_1_^*i*^ and *λ*_2_^*i*^ are weighting parameters. The first and second terms of the right side are the length of the curve and the area of the regions found by contours, respectively. In the level set method, *C* ^*i*^ is represented by the zero level set of *ϕ* ^*i*^ : Ω_*i*_ → *R* ^2^, such that

(8) $$\begin{array}{c} C^{i}=\left\{\left(x,y\right)\in \Omega \;|\;\phi^{i}\left(x,y\right)=0\right\},\\ \Omega_{1}^{i}=\left\{\left(x,y\right)\in \Omega \;|\;\phi^{i}\left(x,y\right)<0\right\},\\ \Omega_{2}^{i}=\left\{\left(x,y\right)\in \Omega \;|\;\phi^{i}\left(x,y\right)>0\right\}. \end{array}$$

The equation for updating level sets is

(9) ∂ϕi∂t=δ(ϕi)(μidiv⁡(∇ϕi|∇ϕi|)−νi) −δ(ϕi)(λ1i(Ii−c1i)2+λ2i(Ii−c2i)2).

Evolve the curve in each channel. Afterwards, a multichannel segmentation combination (MSC) method is used to integrate the information of multiple level sets.

## 3. The Proposed Model

This section develops a robust region-based active contours model for noisy color images segmentation via the narrow band implementation, which segments an image from channel-to-channel. We refer to the proposed model as NBRACM model.

### 3.1. Active Contour Model for Image Segmentation

In order to improve the robustness to strong noise, the proposed model applies local statistics substituting each pixel in the level set functional in the *i*th channel. We define *B*(*x*, *y*) to mask a local region as (3). Therefore, local means in terms of *B*(*x*, *y*) are expressed as

(10) $$\begin{array}{c} u_{x}^{i}=\frac{\int_{\Omega_{y}}B\left(x,y\right)\cdot H\left(\phi^{i}\left(y\right)\right)\cdot I^{i}\left(y\right)dy}{\int_{\Omega_{y}}B\left(x,y\right)\cdot H\left(\phi^{i}\left(y\right)\right)dy},\\ v_{x}^{i}=\frac{\int_{\Omega_{y}}B\left(x,y\right)\cdot \left(1-H\left(\phi^{i}\left(y\right)\right)\right)\cdot I^{i}\left(y\right)dy}{\int_{\Omega_{y}}B\left(x,y\right)\cdot \left(1-H\left(\phi^{i}\left(y\right)\right)\right)dy}. \end{array}$$

Different from the classical CV-like models, the force function is measured by the differences between the local and global intensity means. When a given pixel in the image domain is corrupted by noise, the energy ∫_Ω_(*I*(*x*)−*u*)^2^ *Hdx* + ∫_Ω_(*I*(*x*)−*v*)^2^(1 − *H*)*dx*, which is based on the difference between each pixel and global region means, would reach the minimum even though the curve is not exactly on the boundaries. Hence, some pots in this case are taken wrongly as the object which we are detecting. Problems that appear with the classical CV model derive from the ignorance of local statistics. However, in order to reduce the effect of noise, we replace each pixel with the average of points in its neighborhood. That is, the local information helps to make the proposed model free from noise. Actually, the operation, which replaces each pixel with local region mean, is deemed to approximate the original noisy image with a fitting image which is obtained through neighborhood average filter. In fact, the fitting image would be oversmoothed by the average filter with a lager radius of neighborhood. The lager the radius size is, the fuzzier the image would become. However, when the radius size is small enough, the fitting image is still affected by noise. Similarly, with a smaller neighborhood size, the proposed model using local intensity average will be sensitive to noise. At a larger size, it would be less sensitive to noise and reduce the accuracy of segmentation.

**Figure 1:**
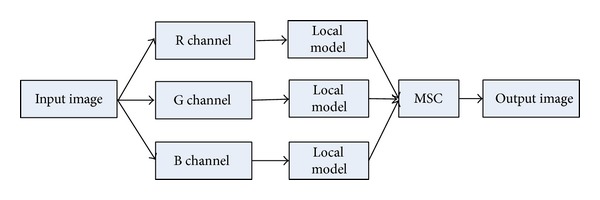
Flowchart of the proposed method.

In this paper, we calculate the force function in each channel; thus, the level set evolution is conducted in each channel separately, that is, the R, G, B channels. As a result, different objects of color image with similar intensities are distinguished easily. Flowchart of the proposed model is shown in Figure 1.

We apply the local region-based active contour model in each channel (R, G, B) of the input image, respectively. After the application of multichannel segmentation combination (MSC) method, we obtain the segmentation results. With the aid of the global intensities means, the internal force in each channel is formed by

(11) Fi(x,H(ϕ(y)))=(ui−uxi)2H(ϕ(y)) +(vi−vxi)2(1−H(ϕ(y))),

where *u* ^*i*^(*x*) and *ν* ^*i*^(*x*) are global means inside and outside of the curve *C* ^*i*^, respectively, which are calculated as

(12) $$\begin{array}{c} u^{i}=\frac{\int_{\Omega_{y}}H\left(\phi^{i}\left(y\right)\right)\cdot I^{i}\left(y\right)dy}{\int_{\Omega_{y}}H\left(\phi^{i}\left(y\right)\right)dy},\\ v^{i}=\frac{\int_{\Omega_{y}}\left(1-H\left(\phi^{i}\left(y\right)\right)\right)\cdot I^{i}\left(y\right)dy}{\int_{\Omega_{y}}\left(1-H\left(\phi^{i}\left(y\right)\right)\right)dy}. \end{array}$$

The *F* ^*i*^ can be substituted directly into (4) to form a completely localized energy within full image domain. To obtain the level set evolution equation for *ϕ* ^*i*^, we take the derivative, expressed as

(13) $$\begin{array}{c} \nabla_{\phi (y)}F^{i}=\delta \left(\phi^{i}\left(y\right)\right)\left({\left(u^{i}-u^{i}\left(x\right)\right)}^{2}-{\left(v^{i}-v^{i}\left(x\right)\right)}^{2}\right). \end{array}$$

By substituting the derivative of *F* ^*i*^ into (5), we obtain the following evolution equation in the full domain implementation:

(14) ∂ϕi∂t(x)=δ(ϕi(x))∫ΩyB(x,y)·δ(ϕi(y))      ×((ui−ui(x))2−(vi−vi(x))2)dy +νδ(ϕi(x))div⁡(∇ϕi(x)|∇ϕi(x)|).

All the partial derivations ∂*ϕ*/∂*x* and ∂*ϕ*/∂*y* in (14) are approximated by the central finite differences. The temporal derivation is discretized as a forward difference. The time dependence *ϕ*(*x*, *y*, *t*) is given in discretized form $\phi_{\begin{array}{c} m,n \end{array}}^{k}$ with spatial index (*m*, *n*) and temporal index *k*. Then, an iteration scheme is obtained by discretization of the partial differential (14):

(15) $$\begin{array}{c} \phi_{m,n}^{k+1}=\phi_{m,n}^{k}+\Delta tL\left(\phi_{m,n}^{k}\right),\;k=0,1,2,\ldots , \end{array}$$

where *L*(*ϕ*_*m*,*n*_^*k*^) is the approximation of the right hand side in the evolution (14).

After the application of the segmenting model in each channel, a combination method should be used to obtain the final results. It is worth noting that the multiple-phase CV [9] model employs two or more level sets. Thus, different sign sequences of the level sets represent different regions. In [14], the level sets are assigned in a different way, namely, one level set per channel. Likewise, in this paper, a set of *N*-size sign sequence can be represented as follows:

(16) S={s=(s1,s2,…,sN) ∣ si=Sign(ϕi),i=1,2,…,N,N∈Z+},

where the functional Sign(*x*) is defined as

(17) $$\begin{array}{c} \text{Sign}\left(x\right)=\{\begin{array}{cc} 1 & \text{if}\;x>0\\ 0 & \text{else}. \end{array} \end{array}$$

**Figure 2:**
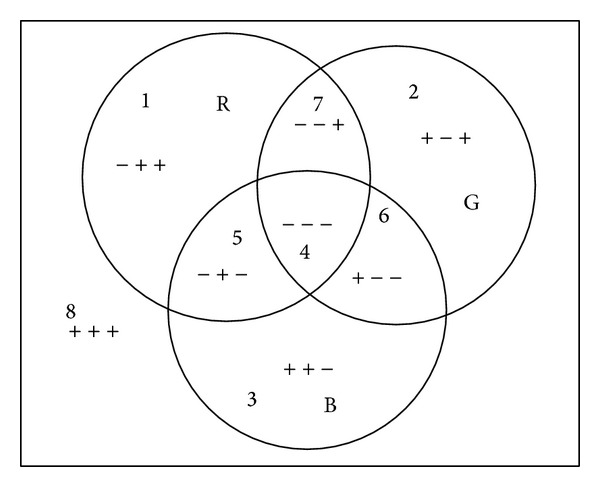
Image for illustrating the MSC method.

Figure 2 employs a simple example to illustrate the MSC method. *ϕ* ^*i*^  (*i* = 1,…, *N*) (*N* = 3 in this paper) are conducted in R, G, B channels, respectively. Each region Ω_*j*_  (*j* = 0,…, 2^*N*^) in the image has a sign array which is composed of *N* signs. In particular, we employ two regions per channel, and hence the proposed model is restricted to be valid only in the case of color images with 2^3^ regions. In this paper, the first sign in the array illustrates whether the corresponding region contains R component. If it is true, it is denoted by a symbol of “−”, and vice versa. The other signs in the array are determined in the same way. For example, the region Ω_4_ in Figure 2 is denoted by “− − −.” Assigning different signs to each region, we can get 2^*N*^  (*N* = 3) regions at most. Thus, we obtain the final segmentation results. However, when the detected regions are more than eight, more level sets for one channel could be used.

### 3.2. Narrow Band Implementation

Throughout the evolution processing, each point in the full image domain with local statistics needs to be computed, which indicates a quite huge computation. Instead of dealing with the entire domain, we only consider an inner and outer band, both sides of the curve, that is, a narrow band around the zero level set. The width of the band should be slightly lager at least than twice as much of the space steps; thus, it ensures a point per side at least. The narrow band implementation is simple, in which the iteration process consists of updating local interior and exterior statistics in the narrow band and performing evolution according to (15).

Firstly, we locate the initial curve with an arbitrary shape and initialize it to be a binary LSF. In the image domain Ω, LSF only takes two values 1 and −1 as

(18) $$\begin{array}{c} \varphi \left(x\right)=\{\begin{array}{cc} -1, & x\in \Omega_{0}\\ 1, & x\in \frac{\Omega }{\Omega_{0}}. \end{array} \end{array}$$

Ω_0_ is the subset of Ω. We should initialize every pixel in the narrow band with the local interior and exterior statistics as well. In addition, when the narrow band moves to include an uninitialized pixel, its local statistics should be initialized as well [10].

Then, the internal force in (13) can be calculated via the narrow band approach:

(19) $$\begin{array}{c} F^{i}\left(x,\phi_{\varepsilon }\right)={\left(u^{i}-u^{i}\left(x\right)\right)}^{2}-{\left(v^{i}-v^{i}\left(x\right)\right)}^{2}. \end{array}$$

Calculating the curvature along the narrow band, we evolve the LSF for every point in the narrow band according to (15) in the *i*th channel until to convergence.

## 4. Implementation and Experimental Results

### 4.1. Implementation

In order to improve efficiency, we only compute values of *ϕ* ^*i*^ in a narrow band around the zero level set [10]. Consequently, we reinitialize *ϕ* ^*i*^ every few iterations using a fast marching scheme [19]. Then the steps of the proposed model are as follows.

1. Locate the initial curve with an arbitrary shape, and initialize it to be a binary LSF. Establish a narrow band around the zero level set in each channel.
2. Initialize every pixel in the narrow band with the local interior and exterior statistics in each channel.
3. Calculate *u* ^*i*^ and *v* ^*i*^ in the global region, *u* ^*i*^(*x*) and *v* ^*i*^(*x*) in the narrow band, and then update the force function according to (19).
4. Get forces from curvature penalty along SDF.
5. For every point in the narrow band, evolve the level set function according to (15).
6. Smooth the SDF using the method in [20].
7. Return to step (3) if the evolution has not converged; otherwise, stop the evolution.
8. Combine the segmentation results of each channel to obtain the final results.

### 4.2. Experimental Results

The performance of the proposed model for noisy color images segmentation has been validated through two groups of experiments carried out on both synthetic and real images. The first group of experiments is conducted to demonstrate the color discrimination and robustness to initial contours. In particular, as for the color discrimination, we apply the proposed model without using the idea from channel-to-channel, which would pick and put the objects with different colors in the same class. The robustness to initial contours of the proposed model is compared with the FMLSM model in [18]. For the second group of experiments, color images added with different noises with different variances are used to show the performance of the proposed model. At the same time, the effect of the radius of local region is also detected. All the experiments are conducted in MATLAB R2010a, on a personal computer with an Intel(R) Core(TM) Duo CPU and 2.00 GB memory.

#### 4.2.1. Color Discrimination

For color images segmentation, the traditional method is to integrate multichannel information, which transforms a color image into a gray one. Actually, our approach can also conduct color image segmentation in the manner mentioned above, called the vectorial NBRACM model. To demonstrate the greater color distinguishing ability of our approach, we conduct experiment on synthetic color image by comparing the vectorial NBRACM with the proposed model, namely, the NBRACM model.

**Figure 3:**
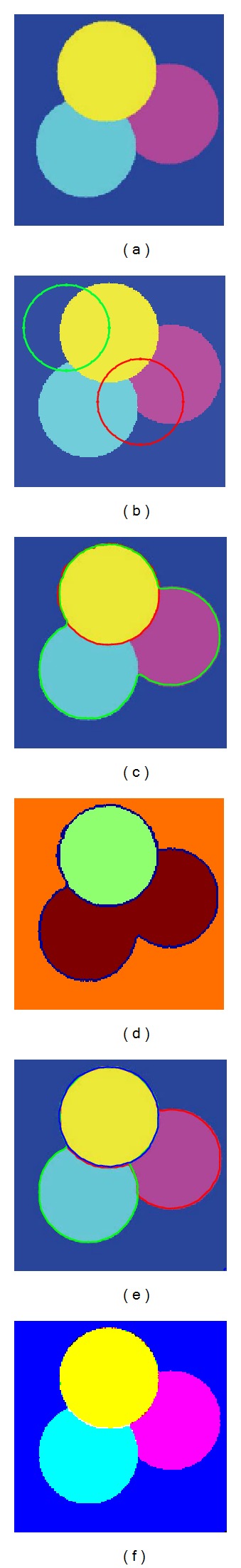
Results for a synthetic image; (a) original image; (b) initial contours; (c) and (d) results of the vectorial NBRACM model; (e) and (f) results of the proposed model.

Figure 3, used in [18], shows a synthetic image with three circles filled with different colors. We choose two circles to be initial contours. The regions, detected by the final contours, are labeled with different colors for easy observation. We use multiple-phase vectorial NBRACM to segment the multiple objects. Figures 3(c) and 3(e) are the corresponding final contours of the vectorial NBRACM and the proposed model, respectively. As we can see, the vectorial NBRACM model can only detect the top yellow ball, taking the bottom objects as the entire one. On the contrary, the proposed model is capable of making a distinction among the three balls.

**Figure 4:**
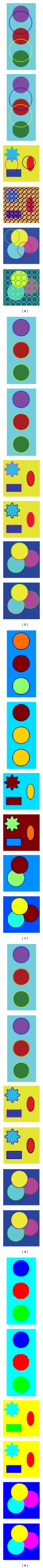
Results for synthetic images with two different initial contours. (a) The two different initial contours; (b) the corresponding final contours of the FMLSM model; (c) the corresponding final labeled images of the FMLSM model; (d) the corresponding final contours of the proposed model; (e) the corresponding final labeled images of the proposed model.

The results for three synthetic images from [18] applied with different initial contours are shown in Figure 4. We compare the FMLSM model in [18] with the proposed model. We choose the initial contours as in [18]. For the first image, when we set two disjoint initial contours as in the first row of Figure 4(a), the two models obtain perfect image segmentation results. When using joint initial contours, for the FMLSM model, the final contours are exactly on the boundaries of the objects. However, the fitting image, which is labeled with different colors, is inaccurate, assigning the bottom two objects with the same color. That is, the bottom objects could not be differentiated by the FMLSM model. However, the proposed model, set with the same initial contours, obtains satisfactory final contours and results. The second synthetic image has three objects with different shapes. When we choose two disjoint circles as initial contours, the same problem happens again. Namely, the dark-blue rectangle and sky-blue star are put in the same class. When the initial contours are set as many small circles, the objects are distinguished well by the FMLSM. On the contrary, our model can differentiate the three objects under these initial conditions. For the third image, the FMLSM obtains accurate segmentation when the initial contours are small circles. The experiments in Figure 4 also demonstrate that the FMLSM obtains satisfactory results when small circles are chosen. However, our model segments the images successfully, showing more robustness to initial contours than the FMLSM model.

Actually, for color images segmentation, both the vectorial NBRACM and the FMLSM [18] models work in the same way. They are similar to the methods which transform the color images into scalar ones [14]. For their multiphase models, there are two active contours evolving in the regions. Both the movements of the contours are determined by the force which averages all the channels using weighted average method. Namely, the intensities of each channel work jointly on the evolving contours during the whole process. However, as can be seen from Figure 1, we assign an active contour to each channel for our model. Thus, the active contours work in each channel independently. The final segmentation results are obtained through MSC method.

To demonstrate the efficiency of our model, we compare the computation CPU time for the above three synthetic images with the vectorial CV model [7], STP-CV model [14], FMLSM [18], and our model (marked as “Ours #2”) in Table 1. In particular, to demonstrate the advantage of narrow band implementation, we also perform the proposed model in the traditional means of computing local statistics in the entire domain (marked as “Ours #1”). As can be seen from Table 1, FMLSM model, which applies split Bregman method, requires the shortest CPU time. On the other hand, our model (#2) is much more efficient than the one using traditional method (#1), which demonstrates the superiority of narrow band implementation. Besides, we can also see that our model is much more efficient than the vectorial CV model, STP-CV model. However, the direction of our future work lies in the incorporation of globally convex image segmentation and the split Bregman method into the proposed model.

**Figure 5:**
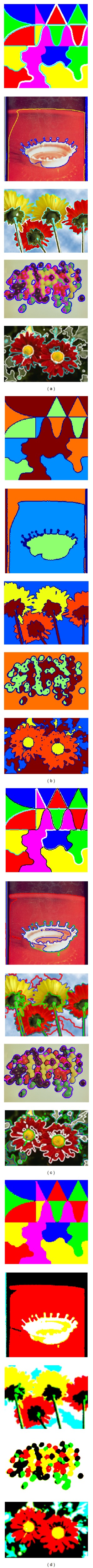
Results for a group of images; (a) the corresponding final contours of FMLSM; (b) the corresponding final segmented images of FMLSM; (c) final contours of the proposed model; (d) results of the proposed model.

The experiments in Figure 5 are conducted on a group of real images. We compare the proposed model with the FMLSM. The image in the first row is a synthetic image with many shapes. The FMLSM model confuses the rose red objects with the blue ones, while our model obtains successful results. The same situation exists in the fourth image. For the flower images, the FMLSM possesses the ability of detecting objects, providing good segmentation. However, benefited from the color discrimination, our model can distinguish more colors, making the segmentation closer to the reality.

#### 4.2.2. Noise Robustness

In this subsection, firstly, we demonstrate that the proposed model possesses more robustness to noise, compared with the multiphase vectorial CV model in [7, 9], the STP-CV model in [14], and FMLSM model in [18]. We choose small circles as initial contours to improve the efficiency.

**Figure 6:**
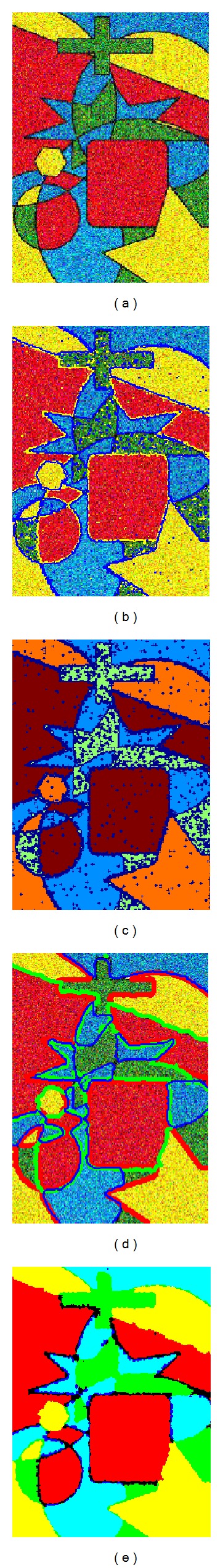
Results for a synthetic image added with Gaussian noise with 0 mean and variance 0.05 using the multiphase vectorial CV model and the proposed model; (a) original noisy image; (b) the corresponding final contours of the multiphase vectorial CV model; (c) the corresponding final labeled image of the multiphase vectorial CV model; (d) the corresponding final contours of the proposed model; (e) the corresponding final labeled image of the proposed model.

Figure 6 shows the segmentation results on a synthetic image added with Gaussian noise with 0 mean and variance 0.05. There are quite many different shapes and four colors in the image. To be fair, we set the same parameters to validate the better performance of the proposed model than that of the multiphase vectorial CV model. As we can see from Figures 6(b)-6(c), the multiphase vectorial CV model detects the boundaries of the objects, dividing them into correct classes. However, when the pixels are polluted by noise, the multiphase vectorial CV model takes some noise pots as the objects. It is due to the fact that the CV-like models are based on the differences between each pixel and the global region means. By contrast, the proposed model is less affected by the noise, providing perfect results as in Figures 6(d)-6(e).

**Figure 7:**
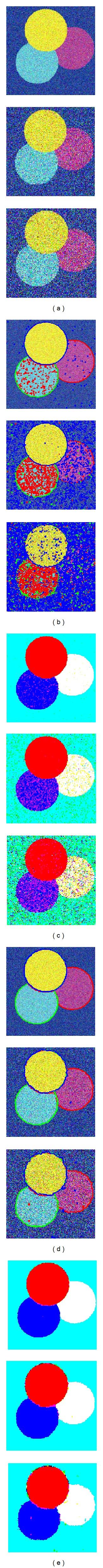
Results for a synthetic image added with Gaussian noise with deviations 0.01, 0.03, and 0.08, respectively. (a) Original noisy images; (b) the final contours of the STP-CV model; (c) the segmentation results labeled with different colors of the STP-CV model; (d) the final contours of the proposed model; (e) segmentation results labeled with different colors of the proposed model.

**Figure 8:**
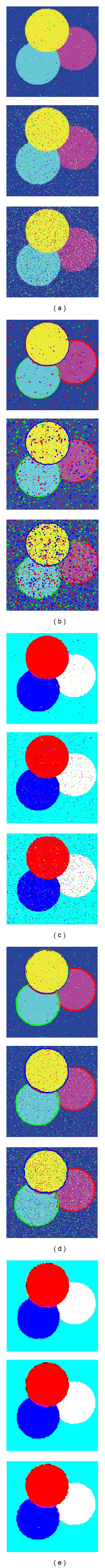
Results for a synthetic image added with salt and pepper noise with deviations 0.01, 0.05, and 0.1, respectively. (a) Original noisy images; (b) the final contours of the STP-CV model; (c) the segmentation results labeled with different colors of the STP-CV model; (d) the final contours of the proposed model; (e) the segmentation results labeled with different colors of the proposed model.

Figures 7 and 8 present the segmentation results of the proposed model and the STP-CV model on a synthetic image. In Figure 7, we add Gaussian noise with mean 0 and variances 0.01, 0.03, and 0.08 to this image, respectively.  Figure 7(a) shows the noise-corrupted images. Figures 7(b)-7(c) show the corresponding final contours and results of the STP-CV model, while Figures 7(d)-7(e) show the corresponding final contours and results of the proposed model. It can be observed from Figure 7(b) that the STP-CV model fails to segment the objects due to the fact that it takes no local statistics into consideration. In this case, the STP-CV model finds not only the objects boundaries but also some noise pots. Also, we set the same parameters for both the STP-CV model and the proposed model for comparison. However, as can be seen from Figures 7(d)-7(e), the proposed model generates more accurate segmentation results. Not only because our model takes local statistics into consideration, but because it possesses the global dependence.

Figure 8 compares the proposed model to the STP-CV model on the synthetic image polluted by salt and pepper noise. We add the salt and pepper noise with variances 0.01, 0.05, and 0.1, respectively, to the synthetic image. Again, a clear improvement is shown. Meanwhile, the STP-CV model shows better performance when dealing with images contaminated by salt and pepper noise than Gaussian noise. Our model, exploiting local intensity information, possesses the ability of segmenting noisy images.

**Figure 9:**
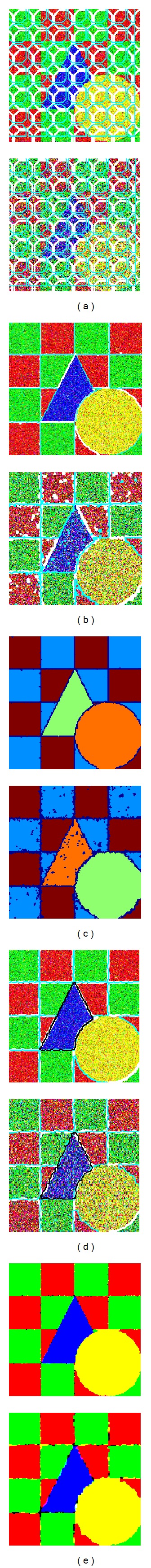
Results for a synthetic image added with Gaussian noise with deviations 0.1, 0.5, respectively. (a) Original noisy images and initial contours; (b) the final contours of the FMLSM model; (c) the segmentation results of the FMLSM model labeled with different colors; (d) the final contours of the proposed model; (e) segmentation results of the proposed model labeled with different colors.

**Figure 10:**
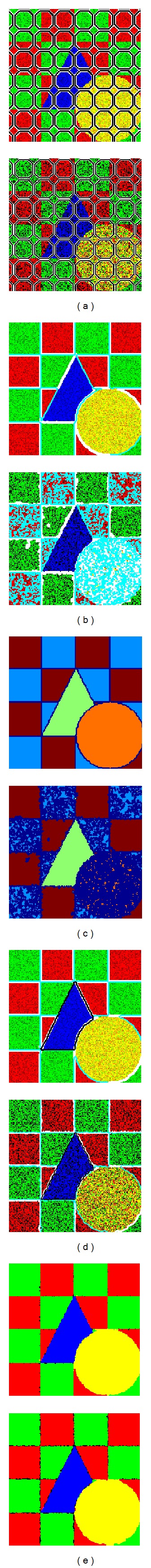
Results for a synthetic image added with multiplicative noise with deviations 0.1, 1, respectively. (a) Original noisy images; (b) the final contours of the FMLSM model; (c) the segmentation results of the FMLSM model labeled with different colors; (d) the final contours of the proposed model; (e) the segmentation results of the proposed model labeled with different colors.

The experiments in Figures 9 and 10 compare the FMLSM with the proposed model in regard to the robustness to various severe noises. A synthetic color image with many objects is employed. In Figure 9, we add severe Gaussian noise with mean 0 and variances 0.1 and 0.5 to the image, respectively. The FMLSM model has the capacity to segment noisy image [18]. Thus, the objects in the noisy image are detected correctly when the variance is 0.1, which can be observed from Figures 9(b)-9(c). However, the FMLSM begins to fail when the variance of noise increases to 0.5. In Figure 10, a similar phenomenon has occurred in the presence of multiplicative noise. Conversely, the proposed model, which employs the local intensities, obtains satisfactory segmentation. The experiments in Figures 9 and 10 show that the proposed model outperforms the FMLSM in the presence of severe noises.

**Figure 11:**
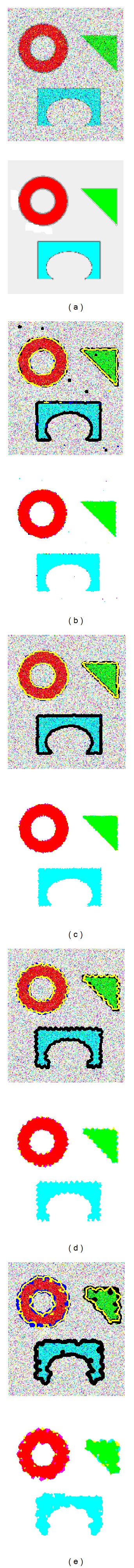
Segmentation results using localizing radii 1, 2, 5, and 9, respectively. (a) Synthetic image with Gaussian noise with variance 0.1 (upper row) and the ground truth image; (b)–(e) the corresponding final results and the segmentation results using radii 1, 2, 5, and 9, respectively.

For the proposed model, the radius of *B*(*x*, *y*) is a vital parameter. If the radius is very small, then the model is sensitive to noise. On the other hand, if the radius of *B*(*x*, *y*) is quite large, the proposed model would have trouble in shape detection. In particular, if the radius grows such that *B*(*x*, *y*) includes the entire image, the local statistics are exactly the global regions statistics; therefore, the differences between them would always be zero. Thus, the proposed model would be invalid. We show the effect of radius size on a synthetic image added with Gaussian noise with variance 0.1 in Figure 11. The radius is set to 1, 2, 5, and 9 pixels, respectively. As can be seen from Figures 11(b)–11(e), with the smallest radius, the objects along with some noise pots are detected. When the radius grows to 9, the detected boundaries are distorted. For quantitative evaluation, four region overlap metrics are used to compare the performances of the models quantitatively. They are the Jaccard similarity (JS) [21], the dice similarity coefficient (Dice) [21, 22], the false positive ratio (rfp), and the false negative ratio (rfn). *S*_1_ represents the foreground of the ground truth image while *S*_2_ stands for the foreground obtained by the models. Since the tested image is a synthetic image, its ground truth has been already known to us. *O* is the common part of *S*_1_ and *S*_2_. *N*(·) indicates the pixel numbers of the region. These metrics are defined as

(20) $$\begin{array}{c} \text{JS}=\frac{N\left(S_{1}\cap S_{2}\right)}{N\left(S_{1}\cup S_{2}\right)},\;\;\text{DSC}=\frac{2N\left(S_{1}\cap S_{2}\right)}{N\left(S_{1}\right)+N\left(S_{2}\right)},\\ \text{RFP}=\frac{N\left(S_{1}\setminus O\right)}{N\left(S_{1}\right)},\;\;\text{RFN}=\frac{N\left(S_{2}\setminus O\right)}{N\left(S_{2}\right)}. \end{array}$$

**Figure 12:**
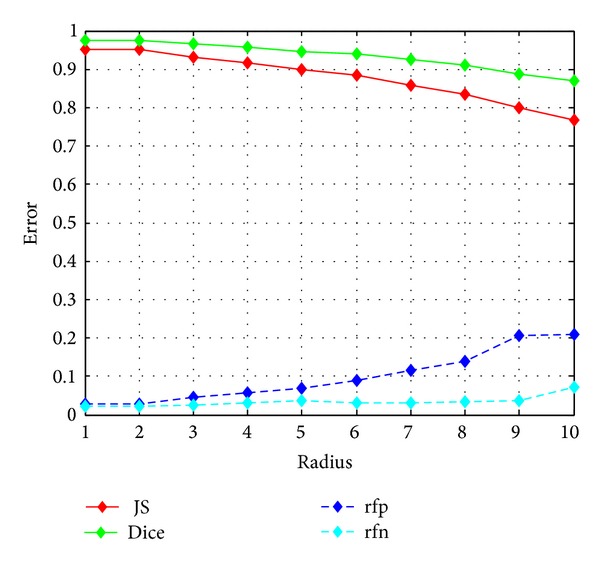
Errors for the segmentations in Figure 11, that is, the JS, Dice, rfp, and rfn with different radius sizes.

The closer the JS and DSC values to 1, and the RFP and RFN values to 0, the better the segmentation results. The radius-error curve is shown in Figure 12.

As seen from Figure 12, the accuracy of the model with the smallest radius is satisfactory. This is due to the fact that though some noise pots are taken wrongly as the objects, their pixel numbers are too little to affect the value of the error. When the radius grows larger, the accuracy becomes lower. Usually, we set radius to be 2 in all experiments in this paper.

**Figure 13:**
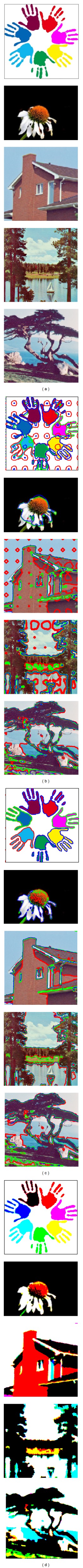
Applications of our model to a group of real images. (a) Original images; (b) intermediate contours; (c) the corresponding final contours; (d) the corresponding segmentation results.

Finally, we apply our model to a group of real images. The results are shown in Figure 13. Again, we can observe that our model can segment these color images well.

## 5. Conclusion

In this paper, a method to segment color images with local region-based active contour model is proposed. The model segments color images from channel-to-channel so that it is able to distinguish close colors. Integrating the merits of the local statistics and the idea of channel-to-channel segmentation, our model possesses the ability of segmenting noisy images, color discrimination, and robustness to noise. After the application of the level set evolution, a MSR method is used to integrate segmentations of each channel. In addition, the proposed model is robust to initial contours. In order to improve efficiency, the level set evolution is calculated in a narrow band frame. Experimental results have demonstrated superior performance of our method.

However, the model cannot cope with images, suffering from intensity inhomogeneity, which is the direction of future work.

## Figures and Tables

**Table 1:** The CPU time in second for the vectorial CV model [7], STP-CV model [14], FMLSM model [18], our model using traditional solution method, and the proposed model for the three synthetic images from Figure 4. The sizes of images are 240 × 110, 170 × 170, and 256 × 256, respectively.

	Vectorial CV	STP-CV	FMLSM	Ours #1	Ours #2
1	50.92	26.72	1.53	50.11	15.83
2	64.52	31.04	1.89	67.13	29.63
3	71.83	39.07	2.01	76.55	32.59

## References

[1] Kurita T, Otsu N, Abdelmalek N (1992). Maximum likelihood thresholding based on population mixture models. *Pattern Recognition*.

[2] Siddiqui FU, Isa NAM (2011). Enhanced moving K-means (EMKM) algorithm for image segmentation. *IEEE Transactions on Consumer Electronics*.

[3] Caselles V, Kimmel R, Sapiro G (1997). Geodesic active contours. *International Journal of Computer Vision*.

[4] Li C, Xu C, Gui C, Fox MD Level set evolution without re-initialization: a new variational formulation.

[5] Li C, Xu C, Gui C, Fox MD (2010). Distance regularized level set evolution and its application to image segmentation. *IEEE Transactions on Image Processing*.

[6] Chan TF, Vese LA (2001). Active contours without edges. *IEEE Transactions on Image Processing*.

[7] Chan TF, Sandberg BY, Vese LA (2000). Active contours without edges for vector-valued images. *Journal of Visual Communication and Image Representation*.

[8] Chan TF, Vese LA An efficient variational multiphase motion for the Mumford-Shah segmentation model.

[9] Vese LA, Chan TF (2002). A multiphase level set framework for image segmentation using the Mumford and Shah model. *International Journal of Computer Vision*.

[10] Lankton S, Tannenbaum A (2008). Localizing region-based active contours. *IEEE Transactions on Image Processing*.

[11] Li C, Kao C, Gore JC, Ding Z (2008). Minimization of region-scalable fitting energy for image segmentation. *IEEE Transactions on Image Processing*.

[12] Zhang K, Song H, Zhang L (2010). Active contours driven by local image fitting energy. *Pattern Recognition*.

[13] Xie X, Wang C, Zhang A, Meng X (2013). Active contours model exploiting hybrid image information: an improved formulation and level set method. *Journal of Computational Information Systems*.

[14] Wei K, Jing ZL, Li YX, Tuo HY (2011). Extended scheme of Chan-Vese models for colour image segmentation. *IET Image Processing*.

[15] Liu L, Zeng L, Shen K, Luan X (2013). Exploiting local intensity information in Chan-Vese model for noisy image segmentation. *Signal Processing*.

[16] Zheng Q, Dong EQ (2013). Narrow band active contour model for local segmentation of medical and texture images. *Acta Automatica Sinica*.

[17] Zheng Y, Li G, Sun X, Zhou X (2009). Fast edge integration based active contours for color images. *Computers & Electrical Engineering*.

[18] Yang Y, Wu B (2012). A new and fast multiphase image segmentation model for color images. *Mathematical Problems in Engineering*.

[19] Sethian JA (1999). *Level Set Methods and Fast Marching Methods: Evolving Interfaces in Computational Geometry, Fluid Mechanics, Computer Vision, and Material Science*.

[20] Sussman M, Smereka P, Osher S (1994). A level set approach for computing solutions to incompressible two-phase flow. *Journal of Computational Physics*.

[21] Vovk U, Pernus F, Likar B (2007). A review of methods for correction of intensity inhomogeneity in MRI. *IEEE Transactions on Medical Imaging*.

[22] Wang L, Li C, Sun Q, Xia D, Kao C (2009). Active contours driven by local and global intensity fitting energy with application to brain MR image segmentation. *Computerized Medical Imaging and Graphics*.

